# Synthesis of Hollow PVP/Ag Nanoparticle Composite Fibers via Electrospinning under a Dense CO_2_ Environment

**DOI:** 10.3390/polym14010089

**Published:** 2021-12-27

**Authors:** Xin Hu, Jiayang He, Li Zhu, Siti Machmudah, Wahyudiono, Hideki Kanda, Motonobu Goto

**Affiliations:** 1 Department of Materials Process Engineering, Nagoya University, Furo-cho, Chikusa-ku, Nagoya 464-8603, Japan; hu.xin@i.mbox.nagoya-u.ac.jp (X.H.); he.jiayang@h.mbox.nagoya-u.ac.jp (J.H.); zhu.li@g.mbox.nagoya-u.ac.jp (L.Z.); wahyudiono@b.mbox.nagoya-u.ac.jp (W.); 2 Department of Chemical Engineering, Institut Teknologi Sepuluh Nopember, Surabaya 60111, Indonesia; machmudah@chem-eng.its.ac.id

**Keywords:** silver nanoparticles, ultrasonic, electrospinning, hollow fibers, dense CO_2_

## Abstract

Polyvinylpyrrolidone (PVP) is used in a wide variety of applications because of its unique chemical and physical features, including its biocompatibility and low toxicity. In this study, hollow PVP/silver nanoparticle (PVP/Ag NP) composite fibers were synthesized. Stable, spherical Ag NPs, with an average size of 14.4 nm, were produced through a facile sonochemical reduction method. A small amount of starch as a potent reducing and stabilizing agent was used during the reduction of Ag ions to Ag NPs. The fabricated Ag NPs were then added to a 10 wt% PVP-dichloromethane (DCM) solution, which was utilized as an electrospinning feed solution under a dense carbon dioxide (CO_2_) environment at 313 K and 5 MPa and an applied voltage of 15 kV. The dense CO_2_ enabled rapid extraction of DCM from the PVP-Ag NPs-DCM solution, which was then dissolved into PVP/Ag NPs, resulting in a hollow structure. Scanning electron microscopy, Fourier-transform infrared (FT-iR) spectroscopy, X-ray diffraction (XRD) and X-ray photoelectron spectroscopy (XPS) analyses, and thermogravimetric analysis (TGA), were used to characterize the electrospinning products.

## 1. Introduction

Silver nanoparticles (Ag NPs) have been used in a wide variety of applications, such as biochemical sensing [1], antibacterial coatings [2], and catalysis [3], because of their unique physicochemical properties, which differ from those of their bulk counterparts. They have been synthesized through numerous methods, such as photochemical methods [4], laser ablation [5], chemical reduction [6], and electrochemical techniques [7]. However, most of these synthesis routes produce low yields of Ag NPs and often require the use of hazardous organic solvents, which pose significant environmental and biological risks.

Sonochemical methods have been utilized for the fabrication of nanomaterials, especially noble material NPs, such as Au [8], Ag [9], and Pt [10]. Recent studies report the use of organic molecules under ultrasonic irradiation due to their comparably excellent reductive capability and fair interaction with the particles, which prevents their oxidation and agglomeration [11]. Starch has been identified as an effective surfactant for the fabrication of Ag NPs in some processes because of its biocompatibility and nontoxicity. Kumar et al. [12] successfully synthesized Ag NPs using starch under ultrasonic irradiation. The formation of spherical Ag NPs using sago starch as a coating agent has also been reported [13].

Electrospinning is a facile technology for fabricating micro- and nanosized polymeric fibers. It has attracted considerable research attention because of its capacity to produce fibers at a large scale. In a conventional electrospinning device, a high-voltage electric area is created between a grounded collector and a spinneret. A syringe pump then feeds the polymer solution via a spinneret into the high-voltage electric area. Hence, the electrostatic force created, rather than the mechanical force, drives the electrospinning process. The synthesized fibers exhibit high surface area-to-volume ratios, which make them suitable as nanoparticle carriers in tissue engineering [14] and controlled-release applications [15] and as electronic sensors [16]. Currently, there has been considerable advancement in the possible applications of hollow fibers in medical applications [17], for gas separation technologies [18], and even in catalysis [19]. Hollow fibers are commonly produced through coaxial capillary or template-assisted methods, where the hollow structure is created through the thermal degradation or selective dissolution of the fiber core. In contrast, we have reported the fabrication of hollow, nano-, and microfibers under sub- and supercritical CO_2_ [20]. Therein, PVP was added to a dichloromethane (DCM) solvent. Subcritical and supercritical CO_2_ acts as a poor solvent for various polymers [21] and can solubilize most organic solvents, including acids, alcohols, and DCM. Therefore, the interaction between the DCM solution and the dense CO_2_ environment caused the generation and growth of the CO_2_-rich voids during the electrospinning process, thereby forming hollow PVP fibers.

In our previous study, Wahyudiono et al. [22,23] have successfully fabricated hollow structure of poly (methyl methacrylate) (PMMA) and PVP by combining the electrospinning with pressurized CO_2_, respectively. To fabricate the hollow structure, several parameters of this process have been optimized. In addition, Ozawa et al. [24] have successfully produced the hollow PVP-TiO_2_ NPs (titanium dioxide nanoparticles) composite fibers. In addition, the isoelectric point of metal oxides varies with the type and size of the metal. For example, as the particle size increases from 6 nm to 104 nm, the isoelectric point of titanium dioxide decreases from 6.0 to 3.8 [25]. However, it is not clear whether the same phenomenon occurs with metal nanoparticles since the metal oxides and metals have different isoelectric points due to the presence or absence of oxygen atoms, which can result in the different interaction with PVP polymers. Hence, we attempted to fabricate hollow PVP-metal nanoparticles composite fibers, something has not been explored so far.

In this study, a novel method for producing hollow PVP/Ag NP composite fibers was reported. Ag NPs were synthesized under ultrasonic irradiation using starch as a stabilizing agent. Then, the produced Ag NPs were added to PVP-DCM, which was the precursor solution for the synthesis of the hollow nano- and microfibers. The properties of PVP, such as high solubility in water and organic solvents, low toxicity, excellent film-forming capabilities, and good binding characteristics, make them suitable for the electrospinning process. The characteristics of the products were investigated. Ag NPs imparted antibacterial properties to the electrospun PVP fibers, making the hollow PVP/Ag NPs composite fibers ideal for biomedical applications [26]. The electrospun PVP fibers have high surface area-to-volume ratios because of their hollow morphology. As a result, more Ag NPs were exposed on the surface, thereby enhancing the antibacterial characteristics of the composite fibers [27].

## 2. Experimental Procedures

### 2.1. Materials

A silver nitrate solution (AgNO_3_, Wako Pure Chemical Industries, Osaka, Japan) was used as the raw material for the ultrasonic process. The starch and distilled water used herein were procured from the same supplier. For the electrospinning process, PVP (MW 1,300,000) (Sigma-Aldrich, Tokyo, Japan) was the solute in the feed solution, whereas DCM (99.0% purity, Wako Pure Chemical Industries) was used as the solvent. Lastly, carbon dioxide (CO_2_, 99% purity) was purchased from Sogo Kariya Sanso, Inc., Kariya, Japan.

### 2.2. Synthesis and Characterization of Ag NPs

**Figure 1:**
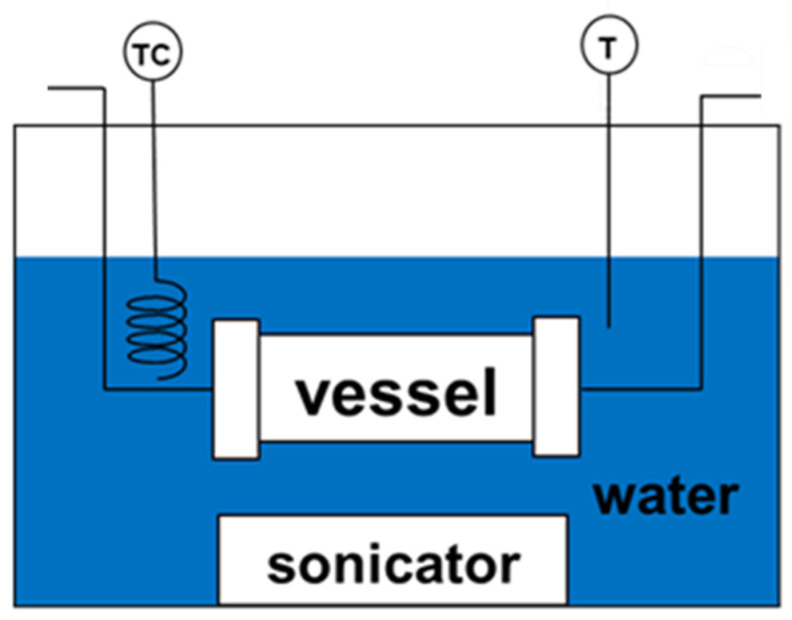
Ultrasonic irradiation apparatus for the synthesis of Ag NPs.

Starch (10 mg) was added to a 10 mL–10 mM AgNO_3_ solution in a glass bottle. To ensure full dissolution of the surfactant, the mixture was stirred and then agitated ultrasonically. Figure 1 shows the schematic of the ultrasonic irradiation apparatus. The mixed solution was put into the stainless steel vessel (TVS-1, 50 mL, Taiatsu Techno Corp., Saitama, Japan). The distance between the vessel and sonicator is approximately 5 cm. The solution was then placed in a water bath maintained at 50 °C. Ultrasonic irradiation was carried out using Ultrasonic Multi Cleaner W–118 (Honda Electronics Company, Toyohashi, Japan). Irradiation was done at 28, 45, 100 kHz, and 600 W (input) for 40, 80, and 120 min.

The optical characteristics of the Ag NPs colloidal solution were investigated using ultraviolet-visible (UV-vis) spectrometry (V-550, JASCO Corporation, Tokyo, Japan). To investigate the morphology and elemental spectra of the produced Ag NPs, transmission electron microscopy (TEM, JEM-2100Plus, JEOL, Tokyo, Japan) with energy-dispersive X-ray spectroscopy (EDX, JED-2300T and Gatan, GIF Quantum ER, JEOL) was done. For such analysis, the Ag NP colloidal solution was dipped onto a TEM grid. From the obtained TEM images, the size distribution of the synthesized Ag NPs was analyzed using ImageJ ver. 1.42, and at least 250 nanoparticles were counted for analysis.

### 2.3. Feed Solution Preparation for Electrospinning

The polymer solution was produced by dissolving 6, 8, and 10 wt% PVP in DCM. Then, a 1 mL Ag NP solution was added to the polymer solution. Prior to this addition, the colloidal Ag NPs solution was freeze-dried using FDU-1200 (EYELA, Tokyo Rikakikai Co, Ltd., Tokyo, Japan).

### 2.4. Synthesis of the Hollow PVP and PVP/Ag NPs Composite Fibers

**Figure 2:**
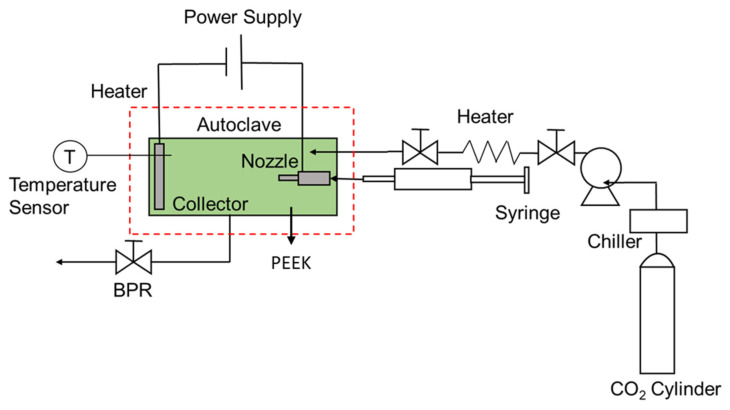
Schematic representation of the electrospinning system.

Figure 2 shows the apparatus used for the electrospinning process, which included a non-conductive polyetheretherketone (PEEK) cylindrical chamber (inner diameter 6 cm; length 20 cm; AKICO, Tokyo, Japan) with cartridge heaters coupled with an electric fan, high-pressure pump (PU–1586, JASCO), back-pressure regulator (BPR) (HPB–450 SUS–316, AKICO), high-pressure syringe pump (PHD–Ultra 4400, Harvard Apparatus, Holliston, MA, USA), high-voltage (HV) power supply unit (HARb–30P1, Matsusada Precision Inc., Tokyo, Japan), and an 8 mL stainless-steel syringe. The nozzle-to-collector distance was 8 cm. The PEEK chamber was heated to 314 ± 3 K. The temperature of the electrospinning process was regulated using a thermocouple, which was installed inside and was put in direct contact with the space of the PEEK vessel. In contrast, K-type thermocouples were installed on the vessel walls to monitor the radial temperature distribution in the PEEK chamber. After the desired temperature was reached, CO_2_ was pumped into the vessel using a PEEK capillary tube to the desired pressure, which was maintained using a BPR. As the necessary conditions were achieved, the polymer feed solution was injected by the high-pressure syringe pump into the chamber through the capillary tube at a flow rate of 0.05 mL/min. Simultaneously, an electrostatic force was generated at 15 kV using the HV power supply. In this device, the polymer solution and CO_2_ were moved separately using a nozzle and were then placed in the stainless-steel flange (anodic electrode). Each experiment was carried out for 20 min and was repeated two to four times to obtain reliable results.

### 2.5. Characterization of the Hollow PVP and PVP/Ag NPs Composite Fibers

The morphology of the produced fibers was examined using SEM (S-4300, Hitachi, Tokyo, Japan) after gold coating (IB-3, Eiko Engineering, Tokyo, Japan). The gold coating was approximately 10 nm thick. ImageJ version 1.42 software was used to determine fiber diameter from the SEM images, and the number of fibers counted for analysis was at least 300. The functional groups on the surfaces of the electrospun fibers generated under each experimental condition were identified through FT-IR spectroscopy (Spectrum Two FT-IR spectrophotometer, PerkinElmer Ltd., Waltham, MA, USA) in the attenuated total reflectance (ATR) mode (golden single reflection ATR system, P/N 10,500 series, Specac) at 4 cm^−1^ resolution from 4000–500 cm^−1^. The structures of the fibers were investigated using X-ray diffraction (XRD; FR–E X-ray diffractometer with Cu Kα radiation (λ = 1.542 Å)). The beam size was approximately 300 μm × 300 μm, whereas the camera length was approximately 70 mm. The fiber samples were placed on a glass substrate and treated with an X-ray beam without additional modifications. The thermal behavior of the electrospun fibers was evaluated using thermogravimetric/differential thermal analyses (TG 8120; Thermo plus, Rigaku, Corp., Tokyo, Japan). Surface elemental compositions of the products were studied using XPS (ESCA-3300, Shimadzu-Kratos, Kyoto, Japan) at a collecting angle of 45° from the average.

## 3. Results and Discussion

### 3.1. Fabrication and Characterization of the Ag NPs Synthesized by Ultrasonic Irradiation

**Figure 3:**
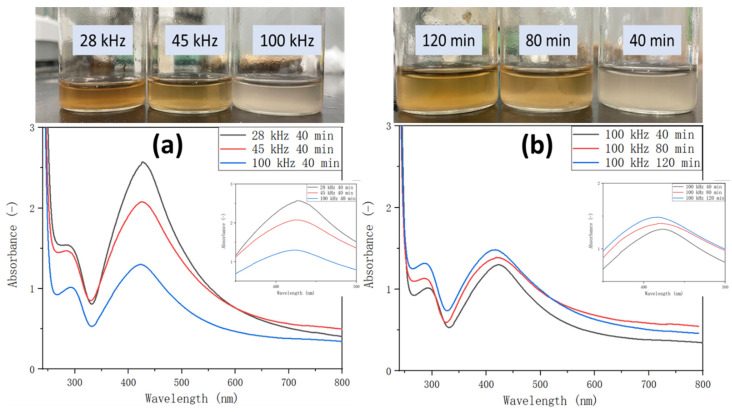
(**a**) UV spectra of the colloidal Ag NP solutions produced through ultrasonic irradiation at different frequencies (28, 45, and 100 kHz) for 40 min sonication duration, and (**b**) different ultrasonic times (40, 80, and 120 min) at frequency of 100 kHz.

The unique optical properties of Ag NPs, that is, the localized surface plasmon resonance (LSPR), are dependent on their shape, diameter, surface structure, and aggregation state, which makes them ideal materials for various applications. Figure 3 shows the UV spectra of the Ag NP solutions fabricated through ultrasonic irradiation at different frequencies and sonication durations. An intense peak at the spectra of the Ag NP solution occurs approximately at 405 nm, which was also observed in the spectra of chemically prepared Ag NPs reported in literature [28]. Figure 3a shows the optical absorbance of the colloidal Ag NP solutions obtained at different ultrasonic frequencies (28, 45, and 100 kHz) and irradiation for 40 min. The color of the Ag NP solution changed from transparent (100 kHz) to yellow (45 kHz), and, finally, to dark brown (28 kHz), which indicates a change in the rate of reduction of AgNO_3_ to Ag NPs [29]. The absorbance of the Ag NP solution decreased as the ultrasonic frequency increased. Furthermore, the intensity of the peak at 405 nm decreased with increasing ultrasonic frequency, which implies that the formation rate of Ag NPs in the colloidal solution decreased with increasing frequency [30]. Previous reports show that the following reactions occur during the sonochemical reduction of metal nanoparticles in the presence of organic additives (reactions 1–4) [31].

(1) H2O → H•+OH• 

(2) 2RH+OH•(H•)→2 R•+H2O(H2) 

(3) $$\mathrm{RH}\;\underset{}{\to }\;\mathrm{radicals}\;\mathrm{and}\;\mathrm{unsteady}\;\mathrm{products}\;$$

(4) Ag(I)+reducing species (H•, R•, etc.)→ Ag(0)

where RH is the organic additive. Reactions (1)–(3) demonstrate the sonochemical fabrication of the reducing radicals and reductants: (1) the sonolysis of water produces ^•^H, (2) the abstraction reaction of RH with ^•^OH or ^•^H produces ^•^R and H_2_, and (3) the pyrolysis of RH and water produces radicals and unsteady compounds. Finally, Ag(I) reduction occurs through many complex reaction steps utilizing the produced reducing species in the previous reactions [32]. Low sonication frequencies can create relatively large bubbles that collapse more forcefully than higher frequencies, thus promoting the generation of more reducing radicals and reductants. Furthermore, low frequencies can motivate collisions between molecules and clusters in the solution, thereby accelerating the reduction of AgNO_3_ to form Ag NPs.

Figure 3b shows the UV spectra of the Ag NP solutions obtained at different ultrasonic times (40, 80, and 120 min) at a frequency of 100 kHz. The nanoparticle solution was transparent after ultrasonication for 40 min. Its color turned yellow after 80 min and darkened after 120 min. The observed transition in the color is possibly due to the change in the Ag NP concentration in the solution with varying ultrasonication durations. Furthermore, the absorbance of the solution increased with increasing ultrasonic time, which corresponds well to the previous conclusion. Longer ultrasonication times imply more time for the reduction of AgNO_3_ to Ag NPs; hence, increasing Ag NP concentration was observed with prolonged sonication. In addition, the LSPR band shifted toward a shorter wavelength area (Figure 3a,b), when the ultrasonic frequency and the sonication time were increased. This shift indicates a reduction in the Ag NP particle size. The mechanism of such phenomenon will be discussed in the next section.

**Figure 4:**
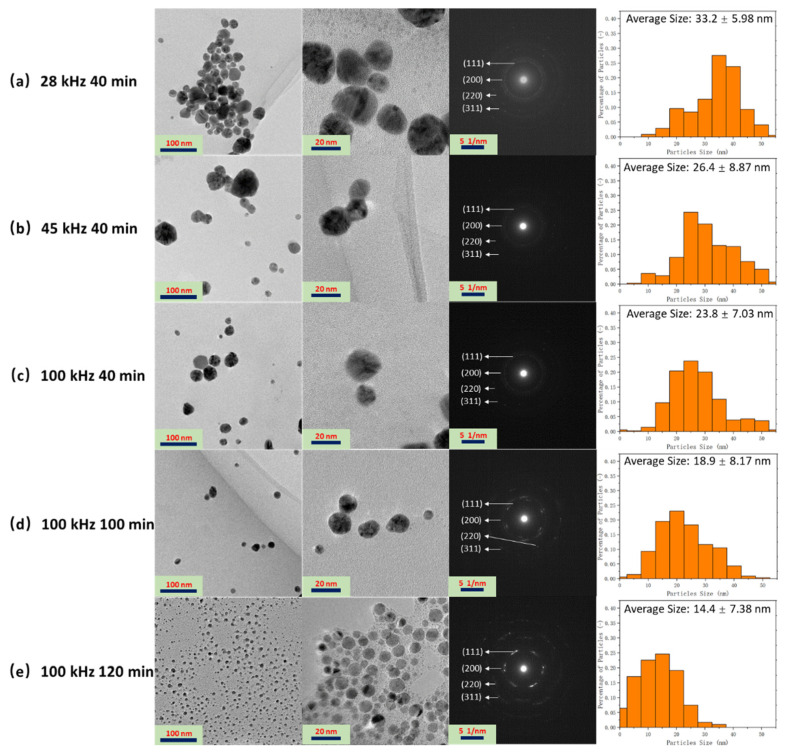
TEM images and SAED patterns of Ag NPs fabricated at different ultrasonic irradiation frequencies (28, 45, and 100 kHz) and durations (40, 80, and 120 min), (**a**) 28 kHz 40 min; (**b**) 45 kHz 40 min; (**c**) 100 kHz 40 min; (**d**) 100 kHz 100 min; (**e**) 100 kHz 120 min.

Figure 4 shows the TEM images, the selected area electron diffraction (SAED) graphs, and size distribution of the Ag NPs fabricated at different ultrasonic irradiation frequencies and times. The synthesized Ag NPs are dispersed and have spheroidal morphologies. The observed diffuse bands in the SAED patterns can be indexed to the (111), (200), (220), and (311) diffraction planes of Ag, which helps determine the phase of the NPs [33]. No discernible differences were observed on changing the ultrasonic parameters, indicating that the frequency and ultrasonic time had no major effect on the crystallinity of the produced Ag NPs. However, ultrasonic parameters were found to have a considerable effect on the size of the nanoparticles. Smaller nanoparticles, by definition, exhibit greater surface area-to-volume ratios, resulting in superior properties. For instance, smaller Ag NPs were reported to have better antibacterial characteristics [34]. The particle size distribution plots show that the size of the fabricated Ag NPs decreased with increasing frequency (Figure 4a–c) and ultrasonic time (Figure 4c–e). Ultrasonic irradiation of the solution generates transient cavitation: bubble formation, growth, and implosive collapse. When a bubble bursts, it creates intense shock waves that spread faster than the speed of sound through the liquid, causing unique sonochemical reactions and high-velocity collisions among the solid particles suspended in the liquid [35]. At 28 kHz ultrasonic frequency, high collisions occurred among the Ag NPs, which resulted in the fusion of the nanoparticles, atoms, or electrons formed from localized melting. This phenomenon can be alleviated at 45 and 100 kHz frequency, hence the decreasing Ag NPs size with increasing ultrasonic frequencies. The findings of this study are in good agreement with those of previous reports. Suslick et al. [36] reported that metal particles that have a mean size of 5–10 μm were aggregated by ultrasonic irradiation at 20 kHz. Okitsu et al. [31] also generated Au NPs using sonochemical reduction of Au (III) ions and found that nanoparticle size decreased with higher ultrasonic frequency during sonication at 20–213 kHz frequencies. In case of ultrasonic time, longer ultrasonic times prevented the aggregation of the NPs, thus reducing the size of the synthesized Ag NPs. A similar finding was reported in the synthesis of zinc oxide NPs through sonication [37].

**Figure 5:**
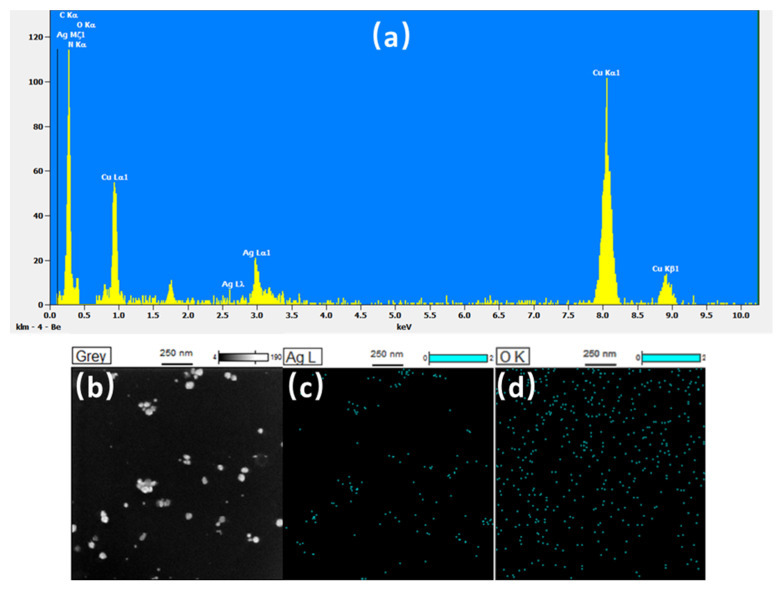
EDX spectra (**a**) and dark-field TEM image of Ag NPs (**b**) with the corresponding EDX map for silver (**c**) and oxygen (**d**) (ultrasonic frequency: 100 kHz, ultrasonic time: 120 min).

Elemental characterization was carried out using EDX analysis. Figure 5a shows the EDX spectra of the Ag NPs synthesized at 100 kHz with sonication for 120 min. Strong signals relating to the elemental silver region (3 keV) in the EDX spectrum indicate the formation of Ag NPs in the solution products. Peaks corresponding to silver, copper (Cu), carbon (C), oxygen (O), and nitrogen (Ni) were also observed in the EDX spectra. The Cu and C peaks observed may be attributed to the TEM grid utilized to load the Ag NPs. In contrast, the peaks corresponding to O and N are attributed to the starch molecule attached to the surface of the Ag NPs and from the AgNO_3_ solution, respectively. Besides, there is an unidentified peak at 1.7 keV in Figure 5a. The characteristics X-ray (keV), which are around 1.7 keV, are Si (1.739 keV), Rb (1.694 keV), Hf (1.644 keV), Ta (1.709 keV) and W (1.774 keV). These elements could not be contained in the sample. The unidentified peak may be caused by the noise or contamination from the sonochemical process.

### 3.2. Fabrication and Characterization of Electrospun PVP Fibers

**Figure 6:**
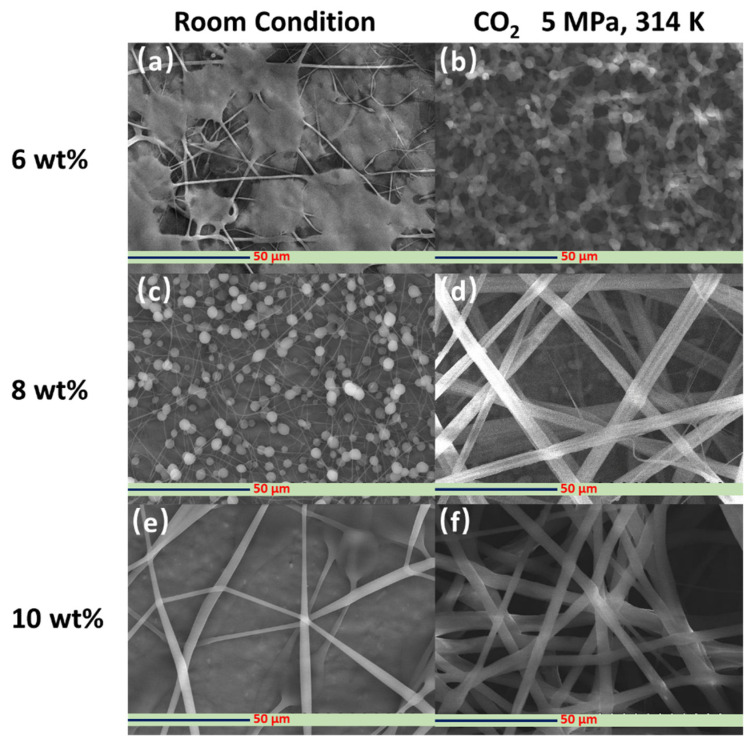
SEM images of the PVP fibers fabricated with (**b**,**d**,**f**) and without (**a**,**c**,**e**) dense CO_2_ at 15 kV at different PVP concentrations (6, 8, and 10 wt%).

Figure 6 shows the SEM images of the PVP fibers synthesized with and without dense CO_2_ at an applied voltage of 15 kV using different concentrations (6, 8, and 10 wt%) of the PVP-DCM solution. Without dense CO_2_, that is, under atmospheric conditions, and at a low polymer solution concentration (6 wt%), the electrospun products showed irregular morphologies and blend (Figure 6a). Furthermore, at the same PVP concentration, almost no solidified electrospun product could be found on the electrospinning collector. The viscosity varies directly with the concentration of polymer, which, in turn, considerably influences the electrohydrodynamic process. Low viscosity can cause the surface tension of the PVP polymer solution to become high. Additionally, incomplete evaporation of the solvent during the electrospinning process occurred when a non-viscous polymer mixture was used as the starting solution, thereby causing the formation of non-solidified, wet electrospun products [38]. In contrast, spherical PVP particles and nascent strings were produced when an 8 wt% PVP polymer solution was utilized (Figure 6c). The increase in the PVP concentration possibly reduced the surface tension and increased the viscosity of the PVP-DCM solution. At 10 wt% PVP, wet fibers were formed on the collector, as seen in Figure 6e.

Under dense CO_2_ environment (pressure: 5 MPa, temperature: 314 K), the electrospun products formed using the 6 wt% PVP solution were mostly nano- and micro-sized spherical particles (Figure 6b). Additionally, almost no PVP polymer strings were synthesized under such operating conditions. Majority of the PVP particles had shriveled morphologies with uniform sizes. These findings imply the rapid evaporation of the solvent, DCM, which resulted in the shrinkage of the PVP particles. By increasing the PVP concentration from 6 to 8 wt% in the presence of dense CO_2_, smooth PVP polymer fibers with nonuniform sizes were fabricated (Figure 6d). These suggest that by changing the PVP concentration and, consequently, the surface tension of the PVP-DCM polymer solution, the morphologies of the electrospun products can be controlled from the accompanied tuning of the evaporation rate of the solvent. A further increase in the PVP concentration to 10 wt% led to the fabrication of smooth, bead-free fibers (Figure 6f). The fibers were straight and appeared to have flat (ribbon-like) morphologies. In addition, the size was more uniform than that of the products fabricated from 8 wt% PVP-DCM solution. Similar results were obtained when the concentration of the PVP solution was changed during the electrospinning process in [39]. The results herein imply that when the concentration of the PVP feeding solution was increased, the entanglement of the PVP chains increased, which promoted the initiation and formation of smooth and uniform-sized electrospun fibers under ambient temperature and a dense CO_2_ environment. Thus, a 10 wt% PVP solution was chosen as the feed solution for the subsequent electrospinning experiments under dense CO_2_.

**Figure 7:**
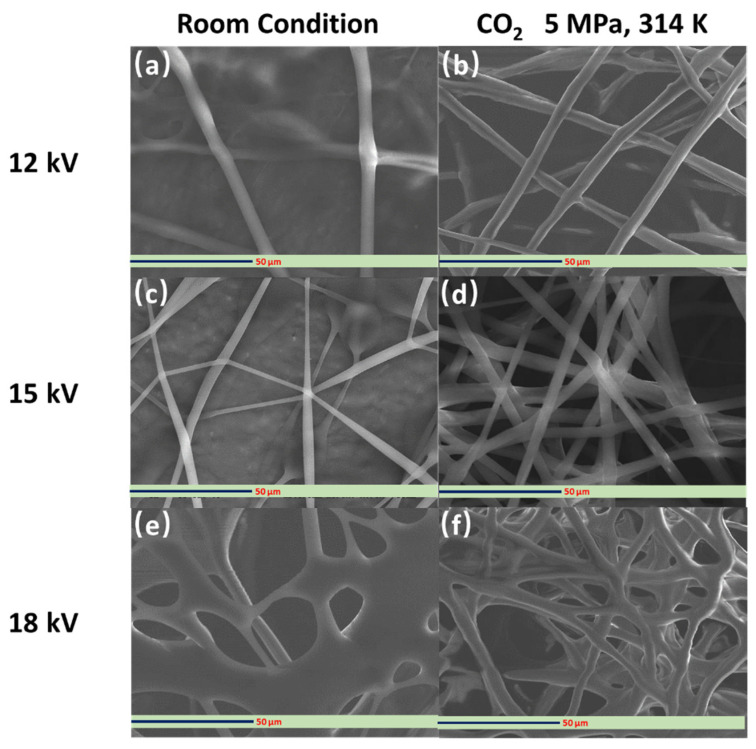
SEM images of PVP fibers fabricated from 10 wt% PVP solution with (**b**,**d**,**f**) and without (**a**,**c**,**e**) dense CO_2_ at various applied voltage (12, 15, and 18 kV).

Figure 7 shows the SEM images of the PVP fibers fabricated using a 10 wt% PVP polymer solution with and without dense CO_2_ at different applied voltages (12, 15, and 18 kV). The applied voltage can determine the intensity of the generated electrical field during the electrospinning process, which significantly affects the characteristics of the PVP solution jet that produces the electrospun fibers. Therefore, the applied voltage is critical for determining the morphology of the electrospun fibers [40]. At higher applied voltages, the stretching stress on the solution jet typically increases, resulting in more delicate fibers. The generation of defects along the fibers is also promoted, increasing the likelihood of bead formation when using an inadequate concentration of the polymer feeding solution [41]. Under room conditions, wet fibers were successfully fabricated under all applied voltage conditions (Figure 7a,c,e). However, under dense CO_2_ conditions, significant differences emerged. As shown in Figure 7b, the fibers fabricated at an applied voltage of 12 kV were not straight. Defects in the fiber direction were also visible. These may be caused by the angle of PVP molecules in the long electrospinning process. At an applied voltage of 18 kV, the electrospun fibers appear to be slightly wet and not straight (Figure 7f), which is possibly caused by the low evaporation rate of DCM as a consequence of shorter electrospinning duration. Olive et al. [42] studied the effect of applied voltage on electrospun fibers and found similar results. Furthermore, the results show that a 15 kV applied voltage was suitable to fabricate the electric field in the electrospinning process (Figure 7d).

### 3.3. Fabrication and Characterization of the Hollow PVP/Ag NP Composite Fibers

**Figure 8:**
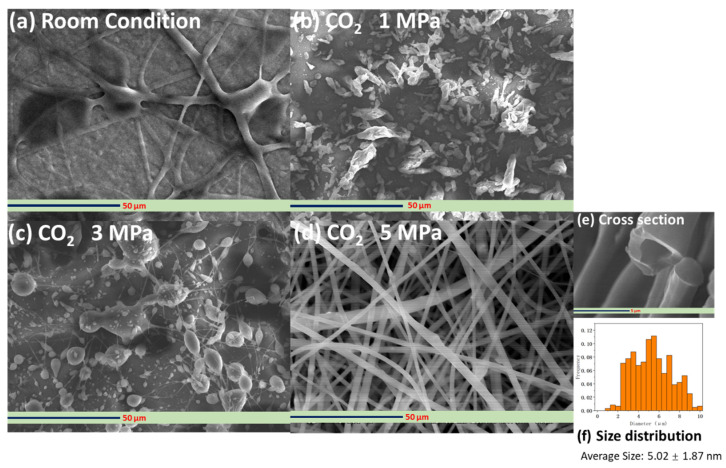
SEM images of the hollow PVP fibers fabricated from PVP solution containing Ag NPs under various CO2 conditions (room conditions, and 1, 3, and 5 MPa), (**a**) Room condition; (**b**) CO_2_ 1MPa; (**c**) CO_2_ 3MPa; (**d**) CO_2_ 5MPa; (**e**) cross section; (**f**) size distribution

Atmospheric parameters, such as temperature and relative humidity, may also impact the morphology of the electrospun fibers in addition to the characteristics of the polymer solution and the factors affecting the electrohydrodynamic process. Figure 8 shows the SEM images of the hollow PVP/Ag NP composite fibers fabricated using the 10 wt% PVP solution under various CO_2_ pressures (room conditions, 1, 3, and 5 MPa) at an applied voltage of 15 kV. Under room conditions, the electrospun products mainly consisted of wet fibers (Figure 8a). At a 1 MPa-CO_2_ pressure, the products are composed solely of nano-and microparticles. No PVP strings are formed (Figure 8b). After increasing the operating pressure to 3 MPa, spherical particles with PVP strings were formed (Figure 8c). At 5 MPa, the products exhibited a uniform, fine fiber morphology (Figure 8d). Bead formation was not observed, which was in agreement with the results of Huang et al. [43]. The average diameter of the electrospun fibers was approximately 5.02 ± 1.87 μm. From the cross-sectional image in Figure 8e, the hollow structure of the fibers can be seen. The formation mechanism of such hollow fibers is as follows. First, the interaction between the polymer solution containing the Ag NPs and CO_2_ may accelerate the rapid evaporation of the solvent (DCM). The phase boundaries then emerge, and, eventually, the electrospun fiber PVP products. Even under supercritical conditions, CO_2_ is an extremely poor solvent for most polymers (including DCM) under the same conditions [44]. As such, superfluous CO_2_ can dissolve into the DCM-rich liquid phase of the PVP polymer solution with Ag NPs. Subsequently, the spinodal decomposition of the PVP-DCM solution might occur, resulting to the generation of a PVP-DCM solution network containing CO_2_-rich bubbles. Such bubbles in the polymer solution might coalesce and expand, forming a jet against the PVP-rich network and propelling PVP-rich phase bubbles radially outward against the inner surface of the jet. This explains the hollow-core-morphology of the electrospun PVP fibers containing Ag NPs. Wahyudiono et al. [45] also reported that hollow PVP fibers containing titanium dioxide (TiO_2_) particles were formed using PVP-DCM containing TiO_2_ NP solution by electrospinning under dense CO_2_. In that report, TiO_2_ NPs were directly added to the PVP-DCM solution. However, because of the low solubility of TiO_2_ in the DCM solution, aggregation between the nanoparticles was observed, resulting in the non-dispersion of the particles on the surface of the fibers and the formation of micro-sized clusters in some areas.

**Figure 9:**
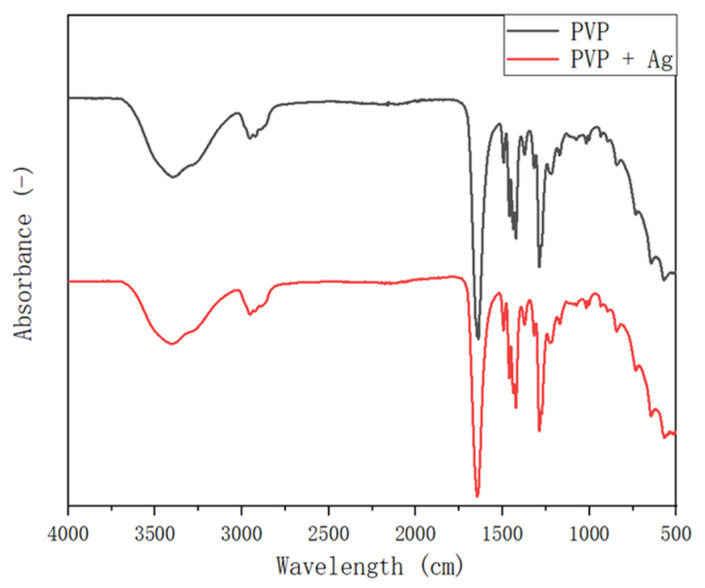
FT-IR spectrum of the pure PVP fibers and of the PVP/Ag NP composite fibers electrospun at 5 MPa and applied voltage of 15 kV.

Figure 9 shows the FT-IR spectra of the pure PVP fibers and of the PVP/Ag NP composite fibers electrospun at 5 MPa and an applied voltage of 15 kV. The peak positions of the infrared bands and functional groups are summarized in Table 1 [24]. Herein, FT-IR spectra were used to analyze the possible structural changes in the PVP molecule, as well as the interaction between PVP and Ag NPs. The synthesized PVP/Ag NP composite fibers share the same FT-IR spectral properties with PVP, suggesting that the products fabricated by electrospinning had almost the same functional groups. Furthermore, this finding suggests that only a small amount of Ag NPs is present in the composite fibers. Hotaby et al. [46] also found a similar result by comparing the FT-IR spectra of pure PVP and PVP-Ag nanocomposite films.

**Figure 10:**
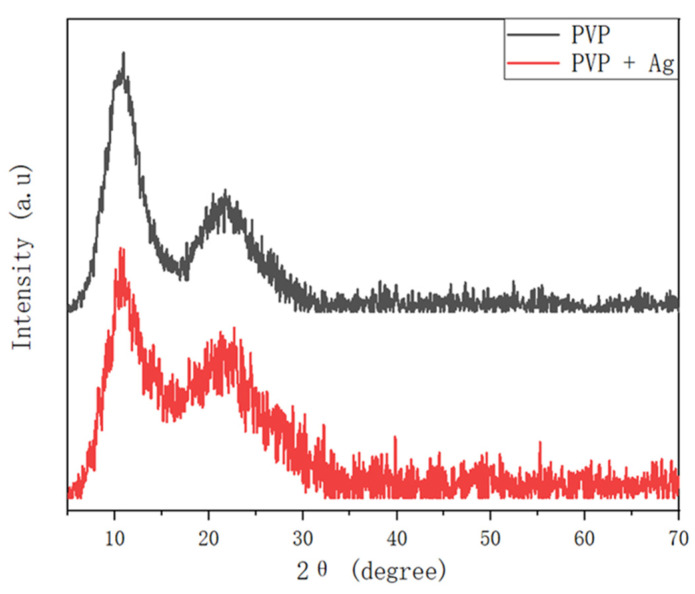
XRD pattern of the pure PVP fibers and of the PVP/Ag NP composite fibers electrospun at 5 MPa at an applied voltage of 15 kV.

Figure 10 shows the XRD patterns of the pure PVP fibers and of the PVP/Ag NP composite fibers electrospun at 5 MPa at an applied voltage of 15 kV. Two diffused halo peaks close to 2θ = 11.78° and 21.94° were observed in the XRD pattern of the pure PVP fiber. This is in good agreement with the results reported by Li et al. [47]. In contrast, the XRD pattern of the PVP/Ag NP composite fibers showed peaks at 2θ = 11.47° and 21.63°. These results also point out the meager amount of Ag NPs in the synthesized PVP/Ag NP composite. These findings may also be due to the masking of the peaks corresponding to Ag NPs by the PVP fibers [46].

**Figure 11:**
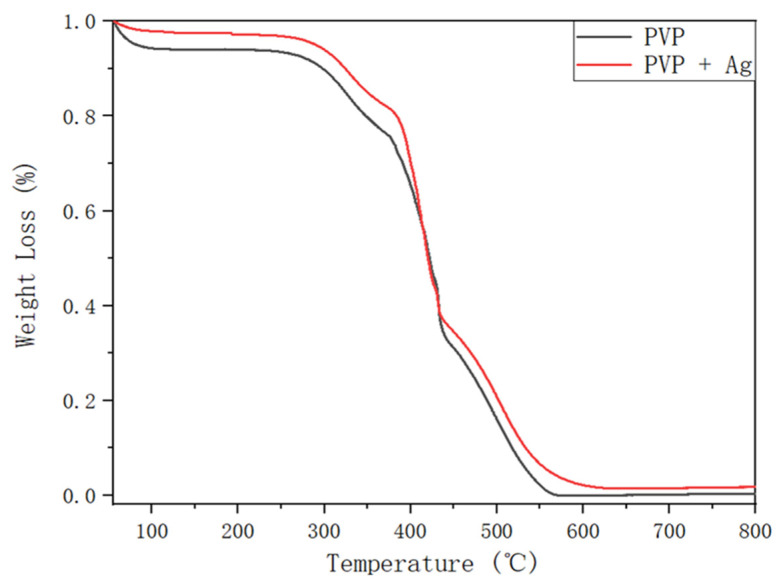
TGA curves of the pure PVP fibers and of the PVP/Ag NP composite fibers electrospun at 5 MPa and applied voltage of 15 kV.

Figure 11 shows the TGA curves of the pure PVP fibers and of the PVP/Ag NP composite fibers (synthesized at 5 MPa and an applied voltage of 15 kV). By measuring the weight variations in response to rising temperature at a constant heating rate, TGA may evaluate a material’s thermal stability and the proportion of the volatile component. Heat transfer and medium diffusion may be used to explain this phenomenon [48]. In this experiment, TGA was carried out at an airflow of 100 mL min^−1^ and a heating rate of 10 °C min^−1^. Pure PVP and electrospun PVP fibers containing Ag NPs were weighed (about 0.6–1 mg) in aluminum pans. After that, the weight loss of these two groups was monitored during the heating process from 40 to 800 °C. The thermal degradation of the pure PVP fibers and of the PVP/Ag NP composite fibers was considered to be the reason for the weight loss observed in Figure 11. During the TG analysis, several sections of loss were observed. The initial weight loss observed at 80 °C is possibly due to the evolution of the volatile compounds from pure PVP and electrospun PVP/Ag NP composite fibers. Thereafter, the thermal deterioration of the electrospun products began at approximately 270 °C. This was followed by a considerable weight loss until 630 °C from the primary devolatilization of the fibers. No further change in weight was observed for both samples beyond this temperature. In general, the polymer matrix–nanoparticle structure can enhance the thermal stability [49]. During the rapid evaporation of DCM, Ag NPs may be dispersed into the PVP matrix. Then, a powerful interaction between PVP and Ag NPs forms through hydrogen bonding in the PVP matrix. Nevertheless, as can be seen in Figure 11, there were no significant differences in the thermal degradation curves of the pure PVP and of the PVP/Ag NP composite fibers possibly due to the low amount of Ag NPs added to the PVP-DCM solution. Consequently, the thermal stability of the PVP/Ag NP composite fibers may not be considerably modified.

**Figure 12:**
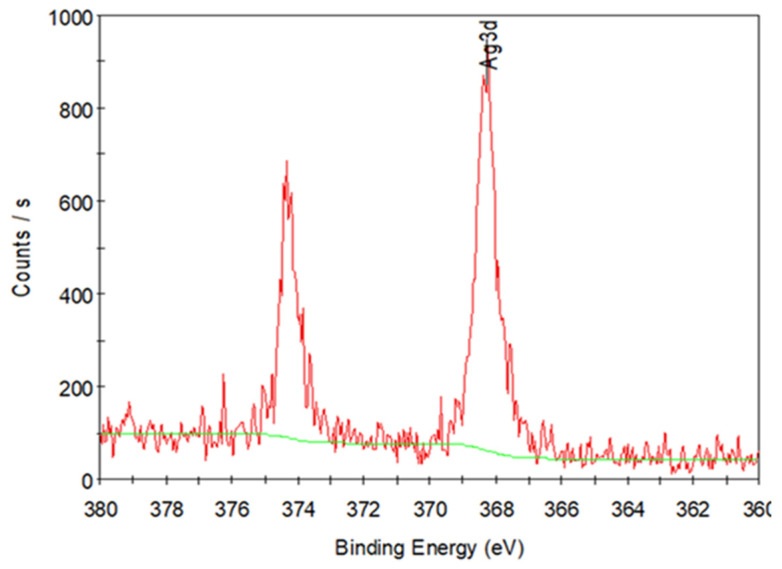
XPS spectra of the residue obtained after the TG analysis of the PVP/Ag NP fibers (5 MPa and applied voltage of 15 kV).

The residue weight (0.025 mg) observed the TG curve of PVP/Ag NP composite fibers (0.73 mg) at temperatures beyond 630 °C reveal the existence of Ag NPs in the electrospun PVP fibers (Figure 11). This permits the separation of the nanoparticles from the polymer, allowing for XPS examination of the sample to acquire precise information on the chemical state of the metal. XPS examination was used after TGA to verify the presence of Ag NPs in the residue. Figure 12 shows the XPS spectra of the residue obtained after the TG analysis of the PVP/Ag NP fibers (5 MPa dense CO_2_ and an applied voltage of 15 kV). Peaks were detected at 368 and 374 eV in the spectra, indicating the presence of Ag NPs in the residue after the TG analysis [50].

## 4. Conclusions

In this study, Ag NPs were initially synthesized through ultrasonic irradiation using starch as a stabilizing and reducing agent of the AgNO_3_ solution. The sonochemical reduction route has an extraordinary potential for generating Ag NPs with a desirable particle size. The ultrasonic frequency and time were found to have a considerable impact on the morphology of the fabricated Ag NPs. In addition, electrospinning of PVP fibers with different PVP concentrations (6, 8, and 10 wt%) at 12–18 kV was investigated under room conditions and dense CO_2_ environment (temperature: 313 K, pressure: 5 MPa). The electrospinning products were compared using SEM images, and suitable parameters for the synthesis of hollow PVP fibers were determined. Thereafter, the electrospinning of PVP-Ag NP fibers was studied at different CO pressures. SEM images revealed that the electrospun PVP/Ag NP composite fibers exhibited the same morphology and hollow structure as the pure PVP fibers. FT-IR spectroscopy and XRD analysis revealed that the pure PVP fibers and PVP/Ag NP composite fibers exhibited similar properties. In addition, XPS confirmed the presence of Ag NPs in the residue obtained after the TG analysis of the hollow PVP/Ag NP composite. Thus, the results showed that polymer fibers with hollow structures containing metal NPs can be fabricated by electrospinning under dense CO_2_.

## Figures and Tables

**Table 1:** Typical bands in infrared spectra of PVP.

Wavenumber (cm^−1^)	Functional Groups
3435.47–3415.45 and 1286.37–1284.84	N–H stretching vibration and C–N stretching vibration from pyrrolidone structure
2950.02–2921.18	C–H stretching for aliphatic compounds
1651.12–1650.46	Carbonyl (C–O) stretching of the five-membered cyclic lactam structure
1493.25–1493.16 and 1461.00–1460.82	C=C aromatic stretching
1422.11–1421.63 and 1372.91–1372.32	C–H bending vibration from methylene groups (aliphatic compound)
843.82–843.29	=C–H bending vibrations (unsaturated compounds)

## Data Availability

The data presented in this study are available on request from the corresponding author.
